# Fabrication and characterization of gold nanoparticles using alginate: *In vitro* and *in vivo* assessment of its administration effects with swimming exercise on diabetic rats

**DOI:** 10.1515/biol-2022-0869

**Published:** 2024-04-17

**Authors:** Vahideh Hashemzadeh, Alireza Hashemzadeh, Reza Mohebbati, Reza Gharari Arefi, Mohammad Ehsan Taghavizadeh Yazdi

**Affiliations:** Department of Sport Science, Binaloud Institute of Higher Education, Mashhad, Iran; Department of Pharmacology, Medicinal Plants, Pharmacological Research Center, Faculty of Medicine, Mashhad University of Medical Sciences, Mashhad, Iran; Department of Physiology, Faculty of Medicine, Gonabad University of Medical Sciences, Gonabad, Iran; Department of Sport Science, Binaloud Institute of Higher Education, Mashhad, Iran; Applied Biomedical Research Center, Mashhad University of Medical Sciences, Mashhad, Iran

**Keywords:** gold nanoparticle, diabetes, oxidative stress, swimming

## Abstract

Gold nanoparticles (AuNPs) have unique features that might lead to the development of a new class of diabetic medicines. AuNPs were biosynthesized utilizing sodium-alginate. UV-Vis-spectroscopy, Fourier transforms infrared, field emission scanning electron microscopy (FESEM), and energy dispersive X-ray were used to examine the particles. The potential of AuNPs for improving the diabetes condition was examined along with swimming in rats. FESEM image revealed the spherical morphology with an average particle size of 106.6 ± 20.8 nm. In the diabetic group, serum glucose, blood urea nitrogen (BUN), creatinine, cholesterol, and triglyceride (TG) levels were significantly higher than the control group. Low-density lipoprotein (LDL) was significantly higher and high-density lipoprotein (HDL) was significantly lower in the diabetic group compared to the control group. Malondialdehyde (MDA) levels were also significantly higher in the D group. However, in the groups treated with swimming and gold, these parameters were significantly improved. Specifically, serum-glucose, BUN, creatinine, cholesterol, and TG levels were significantly reduced, while LDL was significantly decreased in the diabetic + swimming + AuNPs group and HDL was significantly increased in the diabetic + AuNPs group. MDA levels were significantly decreased in the treated groups, and other antioxidants were significantly improved in the diabetic + swimming + AuNPs group. Catalase levels were also significantly improved in the D + gold group. It can be concluded that both AuNPs and swimming can decrease diabetic complications.

## Graphical Abstract

**Figure:**
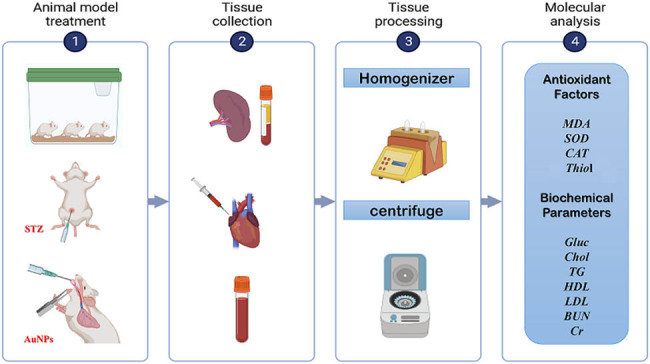

## 1 Introduction

Diabetes is a chronic condition that affects millions of individuals across the world. Diabetes can cause retinopathy, neuropathy, renal failure, and cardiovascular disease, among other complications [1,2]. Type 1 diabetes is brought on by the body’s immune system attacking the pancreatic cells that produce insulin and insulin treatment must last throughout one’s life. When the body develops insulin resistance and the pancreas is unable to generate enough insulin to maintain blood glucose levels within normal ranges, type 2 diabetes develops [3]. Type 2 diabetes is frequently linked to obesity and inactivity [4]. Today, for people with type 1 diabetes and, maybe, some people with type 2 diabetes, insulin therapy is the cornerstone of diabetes care. Blood sugar levels must be regularly assessed during insulin therapy to adjust the dosage. To increase insulin sensitivity and reduce the amount of glucose in the blood, it is required to change lifestyle with regular physical activity and a nutritious diet [5]. The diet should be rich in fruits, vegetables, and whole grains, but low in saturated and Tran’s fats, which can help regulate blood sugar levels more effectively. Physical exercise also lowers the likelihood of heart failure, which is a major diabetic consequence. The drugs metformin sulfonylureas and DPP-4 inhibitors are examples of oral drugs that can reduce blood glucose levels by boosting insulin production or strengthening insulin sensitivity [6]. The agonists of the GLP-1 receptor and SGLT2 blockers are two recent kinds of drugs that could assist in reducing levels of blood glucose by boosting insulin production or decreasing its absorption in the kidneys. These drugs may also help lower the possibility of cardiovascular disease. To achieve optimal blood sugar management, effective diabetes treatment necessitates a collaborative approach involving the patient and the medical professionals [7,8].

Swimming is a type of aerobic activity that has been found to provide a variety of health advantages, including a lower risk of chronic illnesses like diabetes [9–11]. Swimming can help people with diabetes minimize oxidative stress by increasing insulin sensitivity [12]. Insulin resistance, which is related to increased oxidative stress, is a fundamental characteristic of type 2 diabetes. Swimming has been proven to enhance insulin sensitivity in type 2 diabetics, which may aid in the reduction of oxidative stress [13,14]. Swimming has been demonstrated in studies to reduce anxiety-like behavior and oxidative stress in several animal models, as well as to reduce brain oxidative stress, enhance glucose levels, and reduce insulin resistance [15–17]. Swimming may also help to lower oxidative stress by boosting the body’s production of nitric oxide (NO). NO is a chemical that aids in blood flow regulation and inflammation reduction, both of which can contribute to oxidative stress. Swimming has been demonstrated to boost NO generation in people, which may aid in the reduction of oxidative stress and general health [18–20]. Swimming, in addition to reducing oxidative stress, can improve cardiovascular health, lower blood pressure, and promote weight loss in people with diabetes, making it an ideal form of exercise for people with diabetes who may have mobility issues or other health concerns [21,22].

Gold nanoparticles (AuNPs) have demonstrated distinct optical, electrical, and chemical features that enable them to be used in a wide range of applications, including biological studies [23,24]. AuNPs have recently emerged as a viable contender for the treatment of diabetes and might be employed as a novel class of diabetic treatments. AuNPs are particularly capable of interacting with biological molecules due to their high surface area-to-volume ratio [25,26]. AuNPs demonstrated biocompatibility, stability, and ease of modification [27,28]. Due to their anti-inflammatory properties, AuNPs may decrease inflammation in diabetes. Numerous diabetes-related issues, such as nerve damage and renal failure, are greatly influenced by inflammation. By lowering inflammation, AuNPs may be able to help avoid or postpone them. AuNPs were also shown that could decrease blood glucose levels in diabetic rats while having no notable adverse effects [29,30].

AuNPs were able to improve glucose uptake in cells and increase insulin sensitivity [31]. Unlike other antioxidants such as vitamin C or E, AuNPs do not oxidize and do not form toxic byproducts. AuNPs’ morphology and size can also be changed to boost their antioxidant capacity. It has been demonstrated that AuNPs increase the activity of antioxidant enzymes including glutathione peroxidase (GPx) and superoxide dismutase (SOD), which reduce oxidative damage. In addition, it was found that AuNPs could stop lipid peroxidation, a process that can injure cells [32–34].

In general, AuNPs have special qualities that make them an appealing option for biomedical applications, implying that AuNPs have the potential to be employed as an efficient diabetes therapy, while additional study is required to fully appreciate their potential. Herein, alginate-coated AuNPs were prepared in one step and fully characterized. The alginate-coated AuNPs were also tested in a diabetic animal model.

## 2 Materials and methods

The materials used for the synthesis of nanoparticles were procured from Sigma Chemical Group. The synthesized AuNPs were characterized using UV-Vis spectroscopy, Fourier transforms infrared (FTIR) spectroscopy, field emission scanning electron microscopy (FESEM), and energy-dispersive X-ray (EDX) spectroscopy. Also, biochemical assessment kits are purchased from the ParsAzmoon, IR.

### 2.1 Synthesis of AuNPs

In a 250 mL round-bottom flask, 1 g of sodium alginate was dissolved in 90 mL of deionized water. To ensure consistency, the solution was continuously agitated and mixed for a duration of 24 h at room temperature (approximately 20–25°C). Once the alginate was completely dissolved, 2 mL of a 1% gold(iii) chloride trihydrate solution was diluted until the volume reached 10 mL. The gold chloride solution was added dropwise to the flask while being stirred for approximately 5 min. Subsequently, the reaction mixture was heated to facilitate the reduction of gold ions until it reached 80°C. The reaction was continued for 30 min at this temperature. The color of the solution changed from yellow to violet, indicating the formation of AuNPs. Finally, the solution was allowed to cool to room temperature over 2 h. For the supplemental dose, 20 mg/l was orally administered by gavage to rats 1 h before exercise.

### 2.2 Animals

In this research, 35 male Wistar rats aged 8–10 weeks weighing 220 ± 60 g were used. The rats were kept in the animal house of Torbat-e heydarieh University of Medical Sciences. The maintenance conditions included 12 h of light and 12 h of darkness and an ambient temperature of 20–24 degrees. Animals had free access to drinking water during the experimental period. From five days before the start of the experiment and throughout the entire period of the experiment, the rats were fed individually with normal food in separate cages.

Rats were, using a random number table, divided into five experimental groups including the following groups: control, diabetic, diabetic + swimming, diabetic + AuNPs and diabetic + swimming + AuNPs who were treated 8 weeks after diabetes induction. Control group and diabetic group were used as a baseline for comparison, which did not receive any treatment. Diabetic + swimming group has been exercised (swimming exercise). Diabetic + AuNPs group received AuNPs’ supplement. Diabetic + swimming + AuNPs group has been exercised (swimming) and received AuNPs’ supplement.


**Ethical approval:** The research related to animal use has been complied with all the relevant national regulations and institutional policies for the care and use of animals and has been approved by the regional Animal Research Ethics Committee of Working Group of Ethics in Sports Science Research Institute (IR.SSRI.REC.1401.1725).

### 2.3 Diabetes induction

To induce diabetes, one dose (90 mg/kg, BW) of streptozotocin (STZ) (Merck company, Germany) with 0.9% saline solution and under 12-h fasting conditions was administered intraperitoneally to diabetes groups. After administration of STZ injection after 72 hours, to diagnose diabetes of the animals, a few drops of blood were taken from the sinus of the eye in the fasting state, and blood glucose concentration of more than 250 mg/dL was considered diabetic [35].

### 2.4 Swimming method

In the swimming method, 3 h before the experiment, rats were placed in the water tank for 3 min to swim for some time [36].

In the experiment, diabetic and nondiabetic rats after receiving the necessary treatments (20 mg/kg/day, P.O, 1 h before training) individually from the height of 20 cm were placed gently in the water. In these conditions, animals will swim in the water to maintain their stability [37,38]. Then the tested rats were subjected to swimming exercise regularly for 1 h a day during 8 weeks for 5 days [39]. After each exercise, the rats were dried with a soft towel and placed in a storage box near an electric heater for 20 s [40,41].

### 2.5 Measurement of biochemical parameters

Biochemical parameters including serum glucose, cholesterol, triglyceride (TG), high-density lipoprotein (HDL) and low-density lipoprotein (LDL), blood urea nitrogen (BUN), and creatinine were measured by relevant kits (ParsAzmoon, IR) at the end of the experiment (day 56).

### 2.6 Determination of malondialdehyde (MDA)

To analyze for MDA, 0.5 mL of sample was mixed to 1 mL of trichloroacetic acid (10%), 2 ml of HCl, and 1.5 mL of TBA (0.67%). It was then placed in a water bath and boiled for 40 min. A total of 1.5 ml of l-butanol and 0.025 ml of HCl were added to the samples after they had cooled. The following stage involved centrifuging the solution at 1,000 g for 10 min and measuring the supernatant absorbance at 535 nm. The MDA concentration was calculated according to the following equation.

\[\text{MDA}\hspace{.25em}\text{concentration}\hspace{.25em}(\text{M})=\text{Absorbance}/(1.56\times {10}^{5}\hspace{.25em}{\text{cm}}^{-1}\hspace{.25em}{\text{M}}^{-1}).]\]

Our MDA data were expressed per gram of renal tissue.

### 2.7 Measurement of thiol contents

To determine the total thiol concentration, 50 µL of rat renal tissue homogenate was gently mixed in 1 ml Tris-EDTA buffer (pH = 8.6) and the absorbance was read at 412 nm against Tris-EDTA buffer alone (A1). Then, 20 µL of 10 mM solution of DTNB was added to the solution, and it was kept at room temperature for 15 min and the absorbance was read for the second time (A2). The absorbance of the DTNB reagent was also read as blank (B) [42]. The thiol levels were determined by a spectrophotometric method based on the use of Ellman’s reagent, and the results are expressed as per gram of tissue.

\[\text{Total}\hspace{.25em}\text{thiol}\hspace{.25em}\text{concentration}\hspace{.25em}(\text{mM})=(\text{A}2\mbox{--}\text{A}1\mbox{--}\text{B})\times 1.07)/0.05\hspace{14.3em}\times 14,\hspace{-.25em}150.]\]

### 2.8 Catalase (CAT) activity

CAT activity was measured by the Aebi method (1983). This method is based on detection of the rate constant (*k*) (dimension: s − 1, *k*) of hydrogen peroxide destruction by evaluating the reduction in absorbance at 240 nm per minute, and the activity of this enzyme was determined as *K* (rate constant) per liter.

### 2.9 SOD activity

SOD activity was evaluated by the Madesh and Balasubramanian procedure. A colorimetric assay involving generation of superoxide by pyrogallol auto-oxidation and the inhibition of superoxide-dependent reduction of the tetrazolium dye, MTT (3-(4,5-dimethylthiazol-2-yl) 2,5-diphenyltetrazolium bromide), to its Formosan by SOD was measured at 570 nm. An unit of SOD activity was defined based on the amount of enzyme causing 50% inhibition in the MTT reduction rate [43].

### 2.10 Statistical analysis

The data were presented as mean ± standard error of the mean. The Shapiro–Wilk test was used to determine whether the distribution of the data was normal, and after that, the one-way ANOVA and Tukey’s *post hoc* test were used to evaluate the normal data and Mann–Whitney for anormal data. A difference with a *p*-value of 0.05 was taken into account. Statistical analysis was performed using SPSS v11.5.

## 3 Results

### 3.1 UV-Vis spectroscopy and FTIR spectroscopy

**Figure 1:**
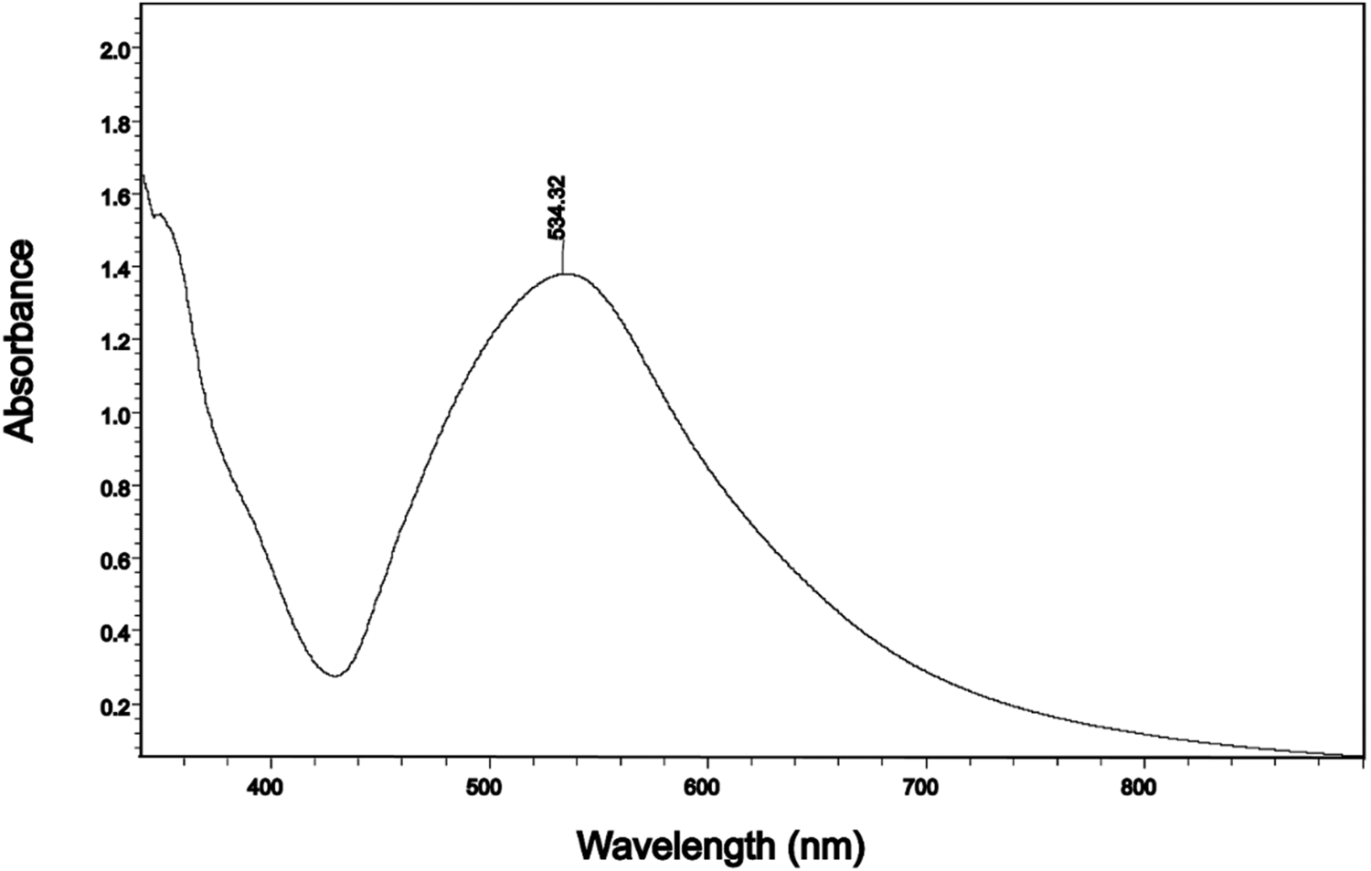
The UV-Vis spectrum of the as-synthesized alginate-coated AuNPs.

The alginate-coated AuNPs’ UV-Vis spectra are shown in Figure 1. The strong absorbance peaks for the alginate-coated AuNPs at 534 nm demonstrated the existence or formation of AuNps.

**Figure 2:**
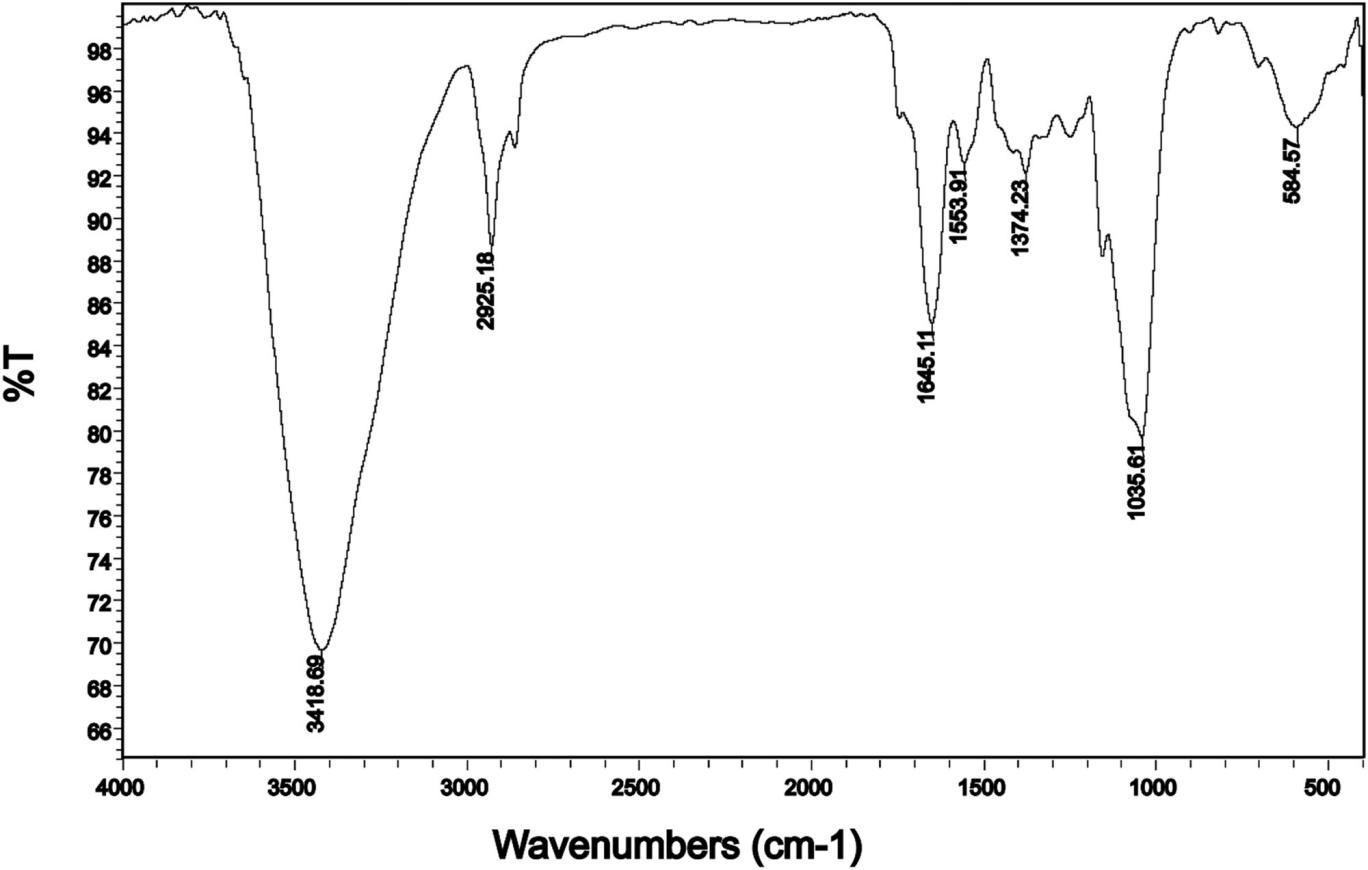
FTIR spectrum of the as-synthesized alginate-coated AuNPs.

The potential functional groups in alginate-coated AuNPs were investigated using FTIR spectroscopy. AuNPs show no adsorption bands in FTIR curves. Hence, the appeared absorption bands were associated with the existing chemical bonds of alginate coating. Figure 2 displays an adsorption band at 3,418 cm^−1^, which was corresponded to the hydroxyl group (^−^OH). The C–H absorption band appeared at 2,925 cm^−1^. The carboxyl group stretching vibrations, the asymmetric and symmetric absorption bands, are observed at 1,645 and 1,418 cm^−1^, respectively. The C–O–C vibration also appeared at 1,035 cm^−1^.

### 3.2 FESEM

**Figure 3:**
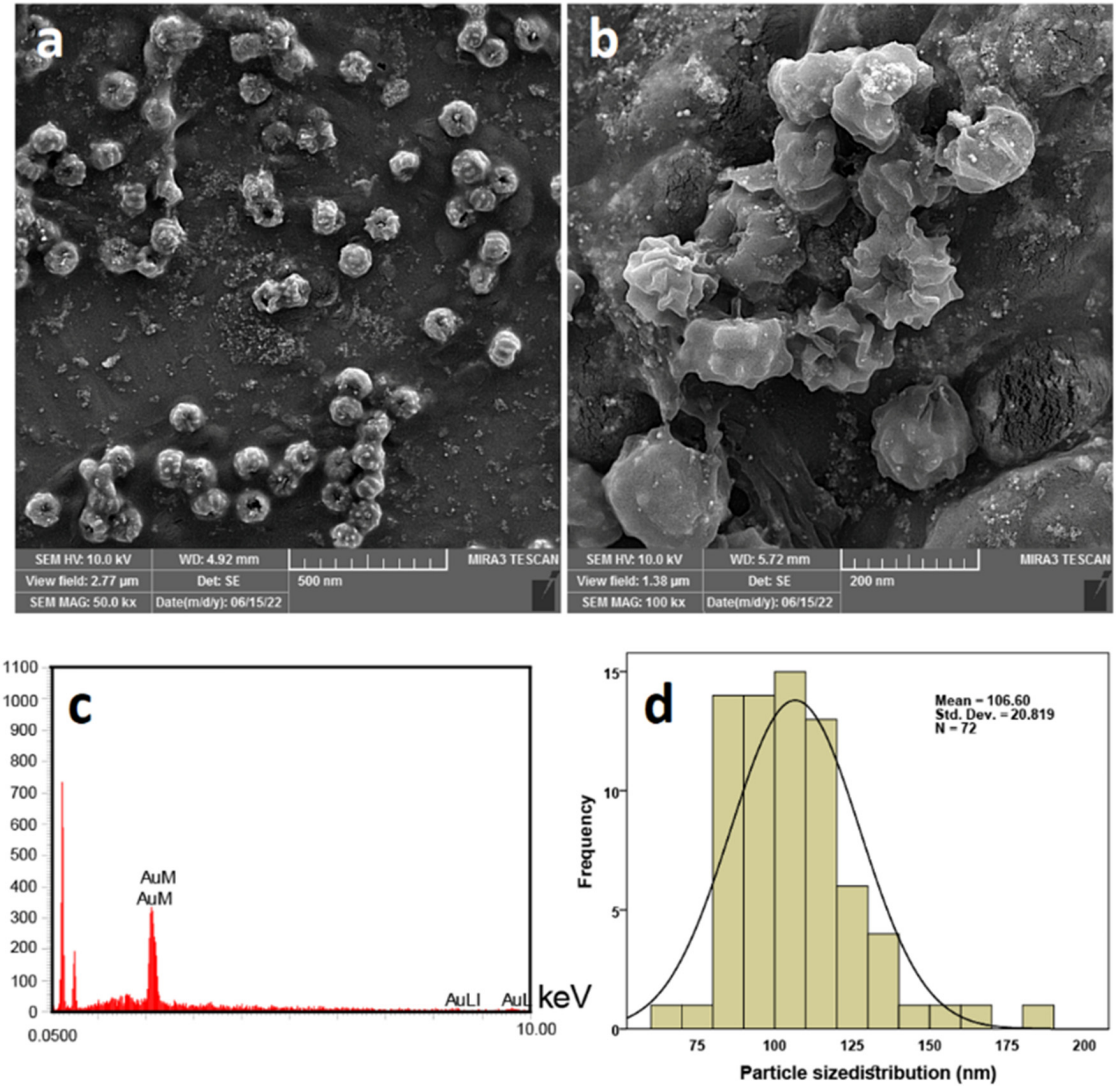
FESEM images (a and b), the EDX analysis (c), and the particle size distribution of the alginate-coated AuNPs (d).

FESEM was used to analyze the morphology and average particle sizes of the alginate-coated AuNPs. It was shown that particles were spherical, and possibly there was a coating of alginate polymer. EDX analyses displayed the purity of the prepared alginate-coated AuNPs, containing gold, carbon, and oxygen. The particle size distribution was also determined by using ImageJ software and SPSS. The mean particle size was 106.6 ± 20.8 nm, indicating a narrow particle size distribution (Figure 3).

### 3.3 Biochemical parameters

**Figure 4:**
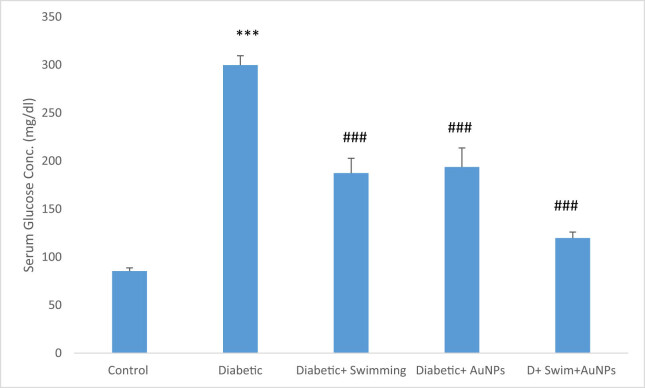
The glucose level in all groups. Data are presented as mean ± SEM. ***p < 0.001 compared to control, ###*p* < 0.001 compared to diabetic group.

Serum glucose in the D group significantly boosted in comparison with the control (*p* < 0.001), while this condition improved in treated groups with exercise and gold (*p* < 0.001) (Figure 4).

**Figure 5:**
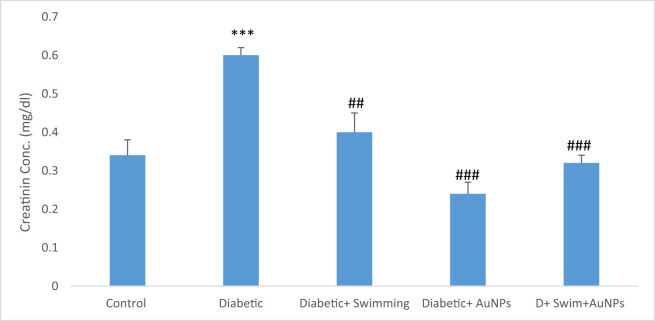
The creatinine level in all groups. Data are presented as mean ± SEM. ***p < 0.001 compared to control, ##*p* < 0.01, ###*p* < 0.001 compared to diabetic group.

**Figure 6:**
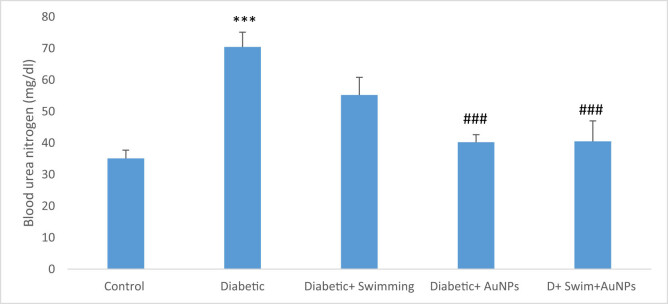
The BUN level in all groups. Data presented as mean ± SEM. ***p < 0.001 compared to control, ##*p* < 0.01, ###*p* < 0.001 compared to diabetic group.

Serum BUN and creatinine significantly increased in the D group compared to the control (*p* < 0.001), while these parameters in the treated groups with exercise and gold significantly reduced (*p* < 0.01 to *p* < 0.001) (Figures 5 and 6).

**Figure 7:**
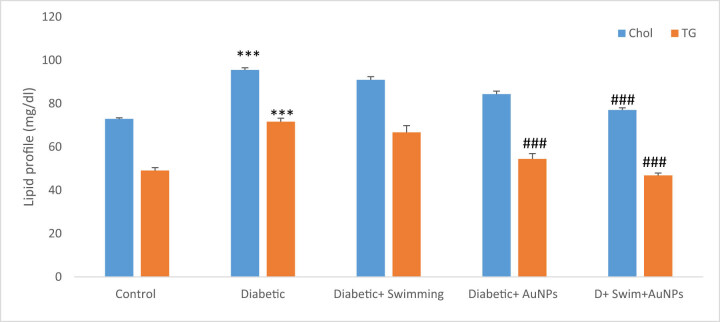
The TG and cholesterol levels in all groups. Data are presented as mean ± SEM. ***p < 0.001 compared to control, ##*p* < 0.01, ###*p* < 0.001 compared to diabetic group.

Also, the cholesterol and TG significantly enhanced in the D group compared to the control (*p* < 0.001), while in treated groups with exercise and gold except D + EX, this condition improved (*p* < 0.001) (Figure 7).

**Figure 8:**
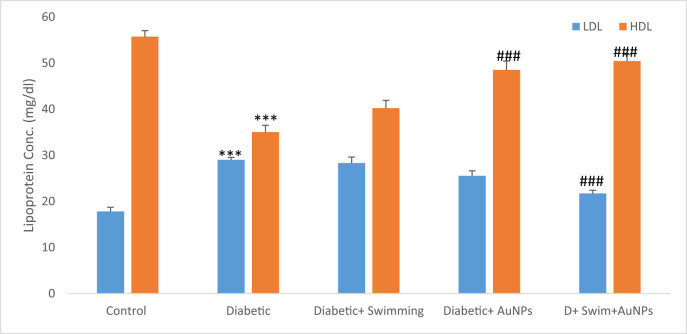
The lipoprotein levels in all groups. Data are presented as mean ± SEM. ***p < 0.001 compared to control, ##*p* < 0.01, ###*p* < 0.001 compared to diabetic group.

Both LDL and HDL significantly changed in the D group than the control (*p* < 0.001) while the LDL significantly decreased in the D + Gold + Ex (*p* < 0.001) and the HDL significantly increased in the D + Gold group (*p* < 0.001) (Figure 8).

MDA in the D group significantly increased than the control (*p* < 0.001), while in treated groups with exercise and gold decreased (*p* < 0.001). Other antioxidants including thiol, SOD, and CAT significantly decreased in the D group than the control (*p* < 0.001), while these reductions were improved in the D + Gold + Ex group. About CAT, improvement also was significant in the D + Gold group than the D group (*p* < 0.001) (Table 1).

**Table 1:** Redox levels in all groups. Data are presented as mean ± SEM

Groups	Parameters			
	MDA	Thiol	SOD	CAT
Control	0.39 ± 0.02	0.64 ± 0.06	0.61 ± 0.06	54.2 ± 4.9
Diabetic	1.43 ± 0.16***	0.09 ± 0.02***	0.07 ± 0.01***	10.3 ± 1.7***
Diabetic + swimming	0.82 ± 0.09###	0.25 ± 0.01	0.13 ± 0.01	32.8 ± 4.5
Diabetic + AuNPs	0.54 ± 0.06###	0.2 ± 0.02	0.21 ± 0.06	46.7 ± 10.5###
D + Swim + AuNPs	0.4 ± 0.03###	0.5 ± 0.06###	0.5 ± 0.03###	41.9 ± 3##

***p < 0.001 compared to control, ##*p* < 0.01, ###*p* < 0.001 compared to diabetic group.

## 4 Discussion

Following the successful production and stabilization of AuNPs using alginate, the potential applications of these nanoparticles in the treatment of diabetes along with the effect of swimming exercise. The alginate-coated AuNPs displayed could enhance biocompatibility, making them suitable for use in biological systems. AuNPs were prepared in deionized water in alginate solutions with the presence of gold(iii) chloride trihydrate. The UV-Vis absorption of Au NPs prepared in alginate has revealed the characteristic peak at 534 nm, suggesting the formation of colloidal AuNPs. The addition of alginate can stabilize small-sized AuNPs and slow down the precipitation rate of large-sized AuNPs. The low stability of AuNPs can complicate their use in biological environments and complicate manufacturing and shipping processes. To counter this, a strategy of drying and dispersion was adopted based on cavities formed by alginate chains to prevent Au NPs from contacting each other. FESEM images showed a spherical shape and an even dispersion of AuNPs with the particle size distribution of 106.6 ± 20.8 nm. The average particle size of 106.6 ± 20.8 nm in AuNPs holds immense potential for biomedical applications. The particle size of AuNPs plays a crucial role in their cellular interactions. Smaller AuNPs (such as those around 106.6 nm) exhibit rapid distribution and renal clearance due to their small size, allowing them to efficiently interact with cells [24,44–46]. These interactions influence cellular uptake, intracellular trafficking, and potential toxicity. The size of AuNPs affects their biocompatibility. Smaller particles may have reduced nonspecific organ accumulation, minimizing potential toxicity [44]. This is crucial for long-term therapeutic applications. The compositional studies (EDX analysis) also showed the purity of the prepared AuNPs. The magnified images of AuNPs show that the particles were bud like and spherical in shape and most probably with a layer of alginate, where AuNPs were contained and protected. The FTIR spectrum also confirmed the presence of alginate in the composition of prepared AuNPs.

The toxicity of metallic nanomaterials is induced by the generation of reactive oxygen species (ROS), which causes oxidative stress [47–49]. AuNPs operate as antioxidants by inhibiting the release of ROS, scavenging free radicals, and increasing the activity of antioxidant enzymes [50,51]. AuNPs exhibit anti-oxidative and anti-hyperglycemic properties, according to *in vivo* investigations. Nano gold with a diameter of 21 nm boosts antioxidant capacity in the blood and liver while decreasing blood glucose levels [52]. AuNPs may induce significant anti-oxidative reactions in biomolecules, such as the introduction into free SH groups, resulting in a reduced antioxidant enzyme profile. The recent study has demonstrated that antioxidants suited to neutralize ROS are effective in reducing experimentally induced diabetes and the degree of diabetic effects [53]. Because of their antioxidant and anti-hyperglycemic effects, AuNPs may be effective in treating diabetes [52,54]. Recently, the inhibitory role of AuNPs on the d-ribose glycation of bovine serum albumin (BSA) revealed that AuNPs of varied sizes and concentrations prevented AGE production in BSA, with a sense of the largest total surface area producing the greatest inhibition. This shows that gold colloidal particles might be used to treat diabetes and hyperglycemia [55]. AuNPs can greatly increase antioxidant production in STZ-induced diabetic rats, a well-known model of type 1 diabetes mellitus. The potential of AuNPs to regulate hyperglycemic situations in diabetic rats was examined in four groups including controls, diabetic group, diabetic group treated with AuNPs, and healthy rats treated with AuNPs. It was discovered that glucose levels in diabetic rats were substantially higher than in controls. Both diabetic and nondiabetic rats have impaired kidney and liver function following AuNP treatment. The results indicated that AuNPs can boost antioxidant activity in STZ-induced diabetic rats [56]. A study also investigated the modulating influence of AuNPs on protective antioxidant mechanism in male Wistar diabetic rats with autism spectrum disorder. Normal littermates that had been fed by control mothers only received injections of citrate buffer [57]. STZ was injected intraperitoneally once into the overnight starved autistic pups to cause diabetes mellitus. In comparison to other metrics, AuNPs at 2.5 mg/kg b. wt. improved several of the stress-related indices (SOD, GPX, and CAT), blood antioxidant capacity, and lipid profile [58]. In another study, diabetic nephropathy exposed human proximal renal tubular epithelial cells HK-2 to glyco-oxidative damage. Antioxidant activity has been demonstrated in AuNPs with a diameter of 30 nm. At physiological pH, the produced AuNPs demonstrated colloidal stability, which reduced high glucose-induced cytotoxicity. The inhibitory effects of AuNPs were blocked by 3-TYP (3-(1*H*-1,2,3-triazol-4-yl) pyridine), a SIRT3 (sirtuin 3) inhibitor. AuNPs could be employed to minimize the risk of diabetic nephropathy development [54]. AuNPs produced using Gymnema Sylvester R. Br have shown a considerable reduction in blood glucose levels in diabetic rats as well as an anti-inflammatory activity, which was measured by serum levels of TNF-, IL-6, and C-reactive protein [59]. *Sambucus nigra* L. (SN) extract-functionalized AuNPs have been found to reduce the amount of glycated hemoglobin while having an anti-inflammatory and antioxidant effect. In an experimental rat model of diabetes, the antidiabetic effects of AuNPs functionalized with SN extract demonstrated that AuNPs lowered MDA levels, COX-2 expression, and proMMP-2 activity while increasing the GSH/GSSG ratio in the muscles and throughout the body and histopathology revealed no morphological abnormalities [52]. AuNPs display strong antioxidant properties that may be used to fight oxidative stress and lessen cellular damage [60–62]. It is possible that they might be used as effective antioxidants in diabetes, where it was observed to reduce oxidative stress and inflammation in animal models of Alzheimer’s and Parkinson’s disease [63–65]. In addition, AuNPs have been used as drug delivery systems in the treatment of cancer, where they may target cancer cells, release therapeutic compounds in a controlled manner [66].

Swimming can assist to lower oxidative stress by boosting the body’s production of antioxidants, which are molecules that neutralize ROS and keep them from causing harm. Swimming exercise has been demonstrated to improve oxidative stress indicators such as MDA, glutathione disulfide (GSSG), and glutathione (GSH) in type 2 diabetic patients [67–69].

An investigation has demonstrated swimming exercise might lessen inflammation and depressive-like behavior in type 2 diabetic mice. Male C57BL6 mice were given a high-fat diet, STZ, and swimming exercises for a period of 4 weeks. According to the findings, mice with type 2 diabetes exhibited considerably more depressive and anhedonia-like behaviors. In type 2 diabetic mice, swimming exercise reduced anhedonia and depressive-like behaviors and also lowered the levels of glucose and inflammatory mediators in the mice’s blood [70]. In another study, the effects of a 6-week swimming program and fenugreek (*Trigonella foenum-graecum*) seed extract on cardiac antioxidant enzyme activity and plasma glucose were examined in STZ-induced diabetic rats [71]. The results indicated that all groups significantly decreased their body weight. Plasma glucose levels were decreased, while cardiac antioxidant enzyme activity significantly increased [72]. Swimming training and *Plantago psyllium* co-administration for 12 weeks improved memory deficit in STZ-nicotinamide-induced type 2 diabetic rats, probably via hypolipidemic and hypoglycemic effects. Another study examined the impact of *Aloe vera* extract and swimming exercise on the lipid profile of rats suffering from diabetes. Results indicated that compared to swimming exercise alone, swimming exercises with *A. vera* extract had a greater impact on changing the lipid profile of rats suffering from diabetes. In addition, compared to swimming training alone, swimming exercise with *A. vera* extract showed a greater effect in improving VLDL, TC, and TG in diabetic rats [73]. Also, swimming exercises with *Dysphania ambrosioides* extract changed the lipid profile of diabetic rats [74]. The goal of our study was to synthesize, identify, and provide AuNPs supplements for measuring antioxidant activities during exercise activities (swimming) in the recovery of rats with type 2 diabetes. It should be highlighted that this was most likely the first time AuNPs’ supplements combined with swimming have been employed in the treatment of type 2 diabetes in an animal model. In this regard, AuNPs with exercise (swimming) in diabetes have benefited the treatment group. As it was anticipated from the aforementioned studies, we expect synergistic effects in the therapy if the AuNPs were used with swimming exercise simultaneously. In this study, the effects of combined swimming and AuNPs on various parameters in STZ-induced diabetic rats have showed that serum glucose levels were significantly higher in the diabetic group compared to the control group, but improved in diabetic + swimming + AuNPs. Diabetes patients are more likely to develop renal disease, which can result in excessive BUN and creatinine levels. This is due to the fact that high blood sugar levels can damage the blood vessels in the kidneys, resulting in a disease known as diabetic nephropathy. Diabetic nephropathy can impair the kidneys’ capacity to filter waste materials from the blood, resulting in elevated BUN and creatinine levels. It is critical for diabetics to frequently assess their kidney function to detect any symptoms of renal impairment early on [75–82]. The result showed that BUN and creatinine levels were likewise considerably higher in the diabetic group, but substantially lower in the groups treated with both swimming and AuNPs, showing the lower possibility of kidney diseases.

Type 2 diabetic patients are more likely to acquire high levels of TGs and cholesterol, which can lead to diabetic dyslipidemia [83–85]. A lack of “good” cholesterol, or HDL, and elevated levels of “bad” cholesterol, or LDL, and TGs describe this syndrome. Diabetic dyslipidemia raises the likelihood of stroke and cardiovascular disease in diabetics. High levels of TGs can also indicate insulin resistance, a risk factor for type 2 diabetes. Insulin resistance occurs when cells in the body grow resistant to insulin’s actions, resulting in high blood sugar levels. This can stimulate the pancreas to generate more insulin, resulting in elevated TG levels [86–88]. Diabetic patients should periodically assess their cholesterol and TG levels and collaborate with their healthcare practitioner to build a treatment plan to maintain these levels. The analyses have revealed that cholesterol and TG levels were higher in the diabetic group, but improved in treatment groups. LDL and HDL values were considerably reduced in the diabetic + swimming + AuNPs group and increased in the diabetic + swimming group. As mentioned, high glucose levels in diabetes can cause oxidative stress and cell damage, leading to consequences such as retinopathy, nephropathy, and neuropathy [89–91]. Hence, the assessment of antioxidant indicators is crucial in diabetes because diabetes is associated with increased oxidative stress, which can cause cell and tissue damage. In this study, MDA levels were greatly reduced in the diabetes group, but higher in the diabetes + swimming + AuNPs group. Other antioxidants, such as total thiol, SOD, and CAT, were also shown to be considerably lower in diabetics. These findings imply that combining swimming with AuNPs has potential therapeutic effects for treating diabetes and its consequences. These findings can be applicable in the real world. Diabetic patients can benefit from swimming for control the diabetic complications. Also, various studies related to the nanoparticle’s applications can open a window to the bright horizon of diabetes treatment. Our study also has some limitations, which are better to considered them in future works including pathological evaluation in various tissues, using different nanoparticle doses for evaluation of the dose dependency manner and assessment of the gene expressions and molecular studies.

## 5 Conclusion

Results of the study indicated the synergic effects of the AuNPs administration in swimmer diabetic rats. Oxidative stress is an important mechanism involved in diabetes in swimmer diabetic rats received AuNPs reduced synergically. Therefore, it can be concluded that both AuNPs and swimming can decrease diabetic complications including biochemical and oxidative markers.
